# Health-Related Quality of Life and Overall Life Satisfaction in People with Serious Mental Illness

**DOI:** 10.1155/2012/245103

**Published:** 2012-11-19

**Authors:** Amy L. Barnes, Meghan E. Murphy, Christopher A. Fowler, Melisa V. Rempfer

**Affiliations:** Department of Psychology, University of Missouri-Kansas City, 5030 Cherry Hall, Kansas City, MO 64110, USA

## Abstract

Quality of life (QoL) in people with schizophrenia and other serious mental illnesses (SMI) is an important outcome goal, yet there is no consistent definition of the construct. We examined three aspects of QoL in persons with SMI: overall life satisfaction, physical health-related QoL (HRQoL), and mental HRQoL. This study had two primary aims: first, to examine whether there are differences in physical and mental HRQoL in persons with SMI, and, second, to investigate the cognitive, clinical, and functional correlates of the three QoL indicators. Participants were 48 persons with SMI who completed assessments of QoL, cognition, functional capacity, psychiatric symptomatology, and medical comorbidity. Results indicate that participants experience similar levels of physical and mental HRQoL, and these two constructs are not related to one another. Physical HRQoL is associated with less medical comorbidity, while mental HRQoL is associated with negative and depressive symptoms. Overall life satisfaction was associated with fewer psychiatric symptoms and less medical comorbidity. This study adds to the important literature defining distinct domains of QoL and supports the necessity of addressing both physical and mental health factors as they relate to recovery and well-being among persons with SMI.

## 1. Introduction

Quality of life (QoL) among persons with schizophrenia and other serious mental illnesses (SMI) has become an important outcome assessment for both research and treatment in recent years [1–4]. However, it remains unclear what factors best predict QoL in this population [3, 5, 6] and a significant challenge for researchers has been the varied methods used to define and measure the broad concept. In an attempt to reduce some of this heterogeneity, research in this area has begun to distinguish between objective and subjective assessments of QoL, as these appear to represent distinct constructs [6]. For instance, objective QoL has been operationalized by examining factors such as the frequency of social interactions, number of hours worked per week, or housing status [5]. However, subjective QoL measurement, which addresses perceived life satisfaction, may be a particularly meaningful treatment goal in this population [4] and is consistent with the recovery model, which emphasizes the lived experience of individuals with SMI [7, 8].

To date, much of the research on life satisfaction in SMI has focused on its relationship with cognitive and clinical symptoms. In terms of cognition, some studies have reported significant correlations between life satisfaction and cognition [9], while others, including a meta-analysis by Tolman and Kurtz [6], have reported either no correlations or inverse correlations between life satisfaction and cognitive functioning. With regard to psychiatric symptomatology, current research has revealed variable relationships. For example, life satisfaction has been associated with psychiatric symptoms, such as depressive and negative symptomatology [8, 10, 11]. However, Narvaez et al. [3] reported that symptom reduction alone did not translate into improved life satisfaction.

Another proposed approach of operationally defining overall life satisfaction in schizophrenia has included examining the impact of disease-related or health-related factors on an individual's perceived QoL, which is referred to as health-related quality of life (HRQoL). HRQoL is a particularly relevant outcome indicator for this population, given that individuals with schizophrenia and other SMIs experience significantly elevated rates of medical comorbidity and early mortality compared to the general population [12]. Thus, the assessment of HRQoL has the benefit of incorporating a broader definition of physical/mental health and its impact on life satisfaction than other general life satisfaction measures, which often have limited coverage of physical health [4, 13, 14]. Previous studies have demonstrated that individuals with schizophrenia and other SMIs report poorer HRQoL than people without SMI [4, 15, 16]. HRQoL in this population has been consistently associated with psychiatric symptomatology, such as depression and general psychopathology [5, 11].

The most widely used measure of HRQoL is the Short Form Health Survey (SF-36), which distinguishes between mental HRQoL (Mental Health Component Summary Scale (MHC)) and physical HRQoL (Physical Health Component Summary Scale (PHC)) [17]. However, few researchers in SMI have begun to examine the distinctions between physical and mental HRQoL [5]. Notably, Meijer et al. [18] reported recently that mental HRQoL, compared to physical HRQoL, was a stronger predictor of overall life satisfaction. In general, little is known about the distinction between mental and physical HRQoL or the relative determinants of each. Given that there are multiple methods for assessing QoL, which have been demonstrated to be distinct from one another [5, 19], further research is needed to understand the unique determinants of the various QoL indicators [5].

The current study simultaneously examined three important aspects of life satisfaction for persons with schizophrenia and other SMIs: overall life satisfaction, mental HRQoL, and physical HRQoL. Specifically, the current study has two aims: (1) to evaluate the subjective concern with physical HRQoL relative to the concern with mental HRQoL and (2) to investigate the cognitive, clinical, and functional correlates of the three QoL indicators.

## 2. Methods

### 2.1. Participants

Forty-eight participants with SMI were recruited from an outpatient treatment program at an urban community mental health center. The Structured Clinical Interview for DSM-IV Disorders (SCID) [20] was used to confirm the diagnosis. Of this sample, 46.9% (*n* = 23) had a SCID-confirmed diagnosis of schizophrenia, 10.2% (*n* = 5) with schizoaffective disorder, 28.6% (*n* = 14) with major depressive disorder, and 12.2% (*n* = 6) with bipolar disorder. One person did not complete the SCID, but diagnosis was confirmed through the chart review and consultation with the treating staff. Exclusionary criteria for the study included known neurological disease, developmental disability, significant sensory/physical impairment that would affect task performance (e.g., blindness), or substance abuse/dependence in the prior 30 days. Additional descriptive information is presented in Table 1.

### 2.2. Procedures

All participants provided an informed consent and all study procedures were approved by the Institutional Review Board at the University of Missouri-Kansas City. Assessments were conducted in a quiet room at the university-affiliated mental health center where participants were enrolled in outpatient psychosocial treatment.

### 2.3. Measures

#### 2.3.1. Health-Related QoL Assessment

The SF-36 [17] is a 36-item measure of subjective health. Widely used in many populations as an HRQoL assessment, the SF-36 contains two primary components, physical health (PHC) and mental health (MHC) [17, 21]. The MHC and PHC share a common standardized metric that utilizes a *t*-distribution ranging from 0 to 100 (*M* = 50, SD = 10). The SF-36 has an extensive evidence supporting its reliability and validity in various populations, including persons with SMI [13, 21–23].

#### 2.3.2. Overall Life Satisfaction

As a measure of overall life satisfaction, we utilized participant scores from item number 23 on the Quality of Life Enjoyment and Satisfaction Questionnaire (Q-LES-Q-23 [24, 25]). This item asks participants to rate their “overall life satisfaction and contentment” in the past week on a 5-point Likert-type scale. This item was selected as the variable of interest, because there are several health-related items on the overall Q-LES-Q measure and we wished to avoid construct overlap with the health-related SF-36 indices. Indeed, this rating of overall life satisfaction and contentment has been found to be distinct from the health domain factor on the Q-LES-Q [25].

#### 2.3.3. Clinical Assessment

The Brief Psychiatric Rating Scale (Expanded) (BPRS-E, [26]) was used to assess the severity of total symptoms, positive symptoms, and negative symptoms. The Hamilton Rating Scale for Depression (HAM-D, [27]) was used to assess the severity of depressive symptoms.

#### 2.3.4. Neurocognitive Assessment

A composite neurocognitive measure was formed using *z*-scores from a battery of 7 measures that are known to be of functional significance in people with SMI [28], including measures of *executive functions* (the Wisconsin Card Sorting Test (WCST-64, [29]), the Trail Making Test, part B (TMT, [30]), and The Controlled Oral Word Association Test (FAS, [31])), *attention/concentration* (the D2 Test of Attention [32]), *processing speed* (the Trail Making Test, part A (TMT A, [30])), *working memory* (Letter-Number Sequencing (LNS, [33])), and *secondary verbal memory* (the California Verbal Learning Test (CVLT-II, [34])).

#### 2.3.5. Functional Capacity

The Brief UCSD Performance-Based Skill Assessment (UPSA-Brief, [35]) was administered as a measure of functional skill capacity. This instrument consists of the finance and communication subscales of the original UCSD Performance-Based Skill Assessment (UPSA, [36]). Validity evidence for the UPSA-Brief has been reported in terms of significant relationships with real-world outcomes [37].

#### 2.3.6. Medical Comorbidity

Participants completed a health checklist indicating if they had ever been diagnosed with any of the 25 listed health problems. Similar to the methods of Dixon et al. [38], an index of the total number of health problems reported by participants was created.

### 2.4. Statistical Analyses

Diagnostic subgroups were combined for all analyses; there were no significant differences among SMI diagnostic groups in terms of age, education, nor any of the study variables. To address our first aim of directly comparing physical HRQoL and mental HRQoL, a paired-sample *t*-test was conducted to examine mean differences in PHC and MHC scores. In addition, we used one sample *t*-test to compare observed PHC and MHC scores in our sample to established population norms for these indices. For our second aim to investigate the cognitive, clinical, and functional correlates of the three QoL indicators, Pearson correlation coefficients were computed to examine the relevant interrelationships.

## 3. Results

Demographic characteristics of the sample are presented in Table 1, while means and standard deviations for the study variables are presented in Table 2.

The first aim of the study was to directly compare physical HRQoL and mental HRQoL. A paired-sample *t*-test revealed no significant difference between mean scores on the PHC (*M* = 43.49) and MHC (*M* = 39.59), *t*(46) = 1.63.

The second aim of this study was to examine the respective correlates of three QoL indicators, physical HRQoL, mental HRQoL, and overall life satisfaction. With regard to correlates of physical HRQoL, the PHC and MHC were not related to one another (*r*(48) = −0.087, *P* > .05), suggesting the participant's subjective concerns with physical health may be independent of their subjective concerns with mental health. As expected, higher physical HRQoL was associated with less medical comorbidity (*r*(48) = −0.518, *P* < .01). Physical HRQoL was not associated with any of the psychiatric, cognitive, or functional measures. See Table 3.

Mental HRQoL was significantly associated with overall life satisfaction (*r*(48) = 0.621, *P* < .01), as well as total symptoms on the BPRS-E (*r*(48) = −0.370,   *P* < .05) and depressive symptomatology (*r*(48) = −.662,  *P* < .01). This association between mental HRQoL and the BPRS-E appears to be driven by the significant relationship between mental HRQoL and negative symptoms (*r*(48) = −.607,   *P* < .01); positive symptoms were not significantly associated with mental HRQoL. In addition, mental HRQoL was not significantly associated with cognitive or functional capacity measures. Better overall life satisfaction was associated with fewer negative (*r*(48) = −.379,   *P* < .01) and depressive (*r*(48) = −.427,   *P* < .01) psychiatric symptoms, as well as less medical comorbidity (*r*(48) = −.339,   *P* < .05). Correlations between overall life satisfaction and cognition and functional capacity did not reach significance (*P* < .10). See Table 3.

## 4. Discussion

The overall purpose of this study was to examine three aspects of subjective QoL in persons with SMI: overall life satisfaction, physical HRQoL, and mental HRQoL. This study differs from the previous QoL research in that it directly compared self-reported physical HRQoL with self-reported mental HRQoL (aim 1). We found no significant difference between physical and mental HRQoL, indicating that participants in this sample were as concerned with their physical health status as they were with mental health status. HRQoL reports in this sample were consistent with those from previous research, which are significantly lower than the general population [4, 39, 40].

Given the significant and well-documented physical health concerns in persons with SMI [12, 38, 41], it is noteworthy that persons with SMI report statistically similar rates of role limitations due to physical and mental health factors. Further, it was interesting to note that although persons are similarly concerned about mental HRQoL and physical HRQoL, there is evidence that these are nonetheless distinct constructs. We found that physical HRQoL and mental HRQoL were not significantly correlated, and with regard to aim 2 each showed distinct patterns of associations with other variables, as described below.

The second aim of this study was to examine patterns of associations between the three distinct QoL indices and measures of clinical, functional, and cognitive status. Physical HRQoL was strongly associated with less medical comorbidity experienced by participants, but not with other variables. This pattern supports the face validity of physical HRQoL and suggests that it is a very specific construct, at least as perceived by respondents with SMI. Similarly, mental HRQoL was associated with psychiatric symptomatology but not with other measures. On the other hand, general life satisfaction showed a broader pattern of correlations and was significantly associated with psychiatric symptoms (total and negative symptoms, and depression), mental HRQoL, and medical comorbidity. Further, there were trend associations for overall life satisfaction with cognitive functioning and functional capacity. These findings are generally consistent with other literature on life satisfaction in this population. Recently, for instance, Meijer et al. [18] reported that depression and anxiety predicted general QoL and that HRQoL (particularly mental HRQoL) was a mediator in this relationship.

Interestingly, however, in our study physical HRQoL was not associated with overall life satisfaction. This finding is in need of further examination in the literature, but one explanation may be that persons with SMI, although concerned about their medical status, do not perceive these health-related limitations as related to other, more general, aspects of their well-being. Given the significant health comorbidities in this population, and the fact that persons with SMI are concerned about their physical HRQoL, further research on this issue is warranted.

Further, our findings highlight the potentiallylimited influence of positive psychiatric symptoms on life satisfaction. In this vein, Lysaker and LaRocco [42] note the importance of not assuming that reductions in QoL are directly related to symptoms of the SMI itself. Perhaps it should come as no surprise that some psychiatric symptoms may have limited influence on overall life satisfaction given that researchers have debated the relative contributions of psychiatric symptoms on other domains of functional outcomes, such as social and community functioning [43].

Our findings should be viewed in light of the associated methodological limitations. Most notably, our study included a relatively small sample size, which may limit the generalizability of our findings to the larger, broad population of persons with SMI. Further, we examined only a subset of the factors that are surely associated with overall life satisfaction. Future studies, with larger samples, are needed that allow for the simultaneous examination of additional social-environmental predictors of QoL, such as trauma history, relationship status, or vocational factors.

An additional limitation is that our findings may be limited to those persons who are actively involved in treatment. Participation within a mental health system may influence some individuals to become more aware of and/or knowledgeable about the influence of factors such as symptoms or medical comorbidities on their functioning. Certainly, as mental health service systems increasingly address physical health issues, one would expect that service consumers would become more attuned to their physical functioning. Thus, our findings may not be consistent with the experiences of those individuals with SMI who are not actively participating in mental health services, or receiving health care in general. Thus, further work is needed to replicate and extend the current findings in other SMI populations, such as younger (and older) participants and those not involved in the formal mental health system.

## 5. Conclusion

This study adds to the important line of research on overall life satisfaction, which ultimately may have important policy and clinical implications in this population. A growing body of outcomes research has revealed that improvements in cognition, reduction of psychiatric symptoms, and access to social services may be necessary, but not sufficient methods of improving life satisfaction in people with SMI [6, 44]. Our findings that persons with SMI are concerned with both mental and physical HRQoL and that various aspects of QoL have distinct patterns of association indicate that recovery and improvements in well-being require attention to multiple levels of factors, including medical, psychiatric, and psychosocial ones. These findings therefore support the growing emphasis on more integrated mental and physical health services, particularly for populations such as persons with SMI who have significant health comorbidities. The appreciation of the physical health care needs of individuals within the mental health system is consistent with emerging state and national trends, which advocate for addressing both mental health and physical health as a part of a whole-body approach to well-being [45].

## Figures and Tables

**Table 1:** Descriptive characteristics of the sample.

Total *N* = 48	*N* (%)
Gender	
Male	25 (52%)
Female	23 (48%)
Race/ethnicity	
African American	30 (62.5%)
Caucasian	13 (27.1%)
Multiracial	5 (10.4%)
Marital status	
Never married	23 (47.9%)
Divorced/widowed/separated	18 (37.5%)
Married/living together	7 (14.6%)
Education	
Less than high school	17 (35.4%)
High school/GED	13 (27.1%)
Posthigh school/no college	1 (2.1%)
Some college	16 (33.3%)
College beyond 4 year degree	1 (2.1%)
Living situation	
Lives independently	31 (64.6%)
Lives with relatives	7 (14.6%)
Supervised care housing	6 (12.5%)
Long-term care facility	1 (2.1%)
Homeless	3 (6.3%)

**Table 2:** Means and standard deviations.

Total *N* = 48	Mean (SD)
PHC	43.49 (9.97)
MHC	39.59 (12.20)
Life satisfaction	3.50 (1.05)
BPRS-E (total score)	45.67 (10.77)
Positive symptoms	1.68 (0.63/range 1–4)
Negative symptoms	2.32 (0.79/range 1–4)
HAM-D	20.02 (10.87)
UPSA-B	70.43 (14.65)
Medical comorbidity	5.27 (3.43)

Note: PHC: physical health component of the SF-36, MHC: mental health component of the SF-36, BPRS-E: brief psychiatric rating scale (expanded), HAM-D: hamilton depression rating scale, and UPSA-B: University of California San Diego performance-based skills assessment (brief).

**Table 3:** Summary of bivariate correlations among quality of life indicators and psychiatric symptoms, cognition, functional capacity, and medical comorbidity.

	PHC	MHC	Life satisfaction
PHC		−0.087	0.159
MHC			0.621**
BPRS-E (total score)	−0.079	−0.370*	−0.306*
Positive symptoms	0.070	−0.016	−0.058
Negative symptoms	−0.038	−0.607**	−0.379**
HAM-D	0.071	−0.662**	−0.427**
Cognitive composite	0.014	0.094	−0.281^+^
UPSA-B	0.044	−0.024	−0.243^+^
Medical comorbidity	−0.518**	−0.262^+^	−0.339*

Note: PHC: physical health component of the SF-36, MHC: mental health component of the SF-36, BPRS-E: brief psychiatric rating scale (expanded), HAM-D: Hamilton depression rating scale, and UPSA-B: University of California San Diego performance-based skills assessment (brief). *P is significant at the 0.05 level (two tailed), **P is significant at the 0.01 level (two tailed), and ^+^ *P* < 0.10.
